# Supplementary material for: Sphingosine-1-Phosphate-derived 2-Hexadecenal is a central mediator of ocular neovascularization by inhibiting Sphingosine-1-Phosphate receptor 5
Source: Nat Commun. 2026 Apr 14;17:3488. doi: 10.1038/s41467-026-71792-3 (PMC13079744; doi:10.1038/s41467-026-71792-3)
Supplement: Supplementary file 1 — Supplementary Information [file 41467_2026_71792_MOESM1_ESM.pdf]

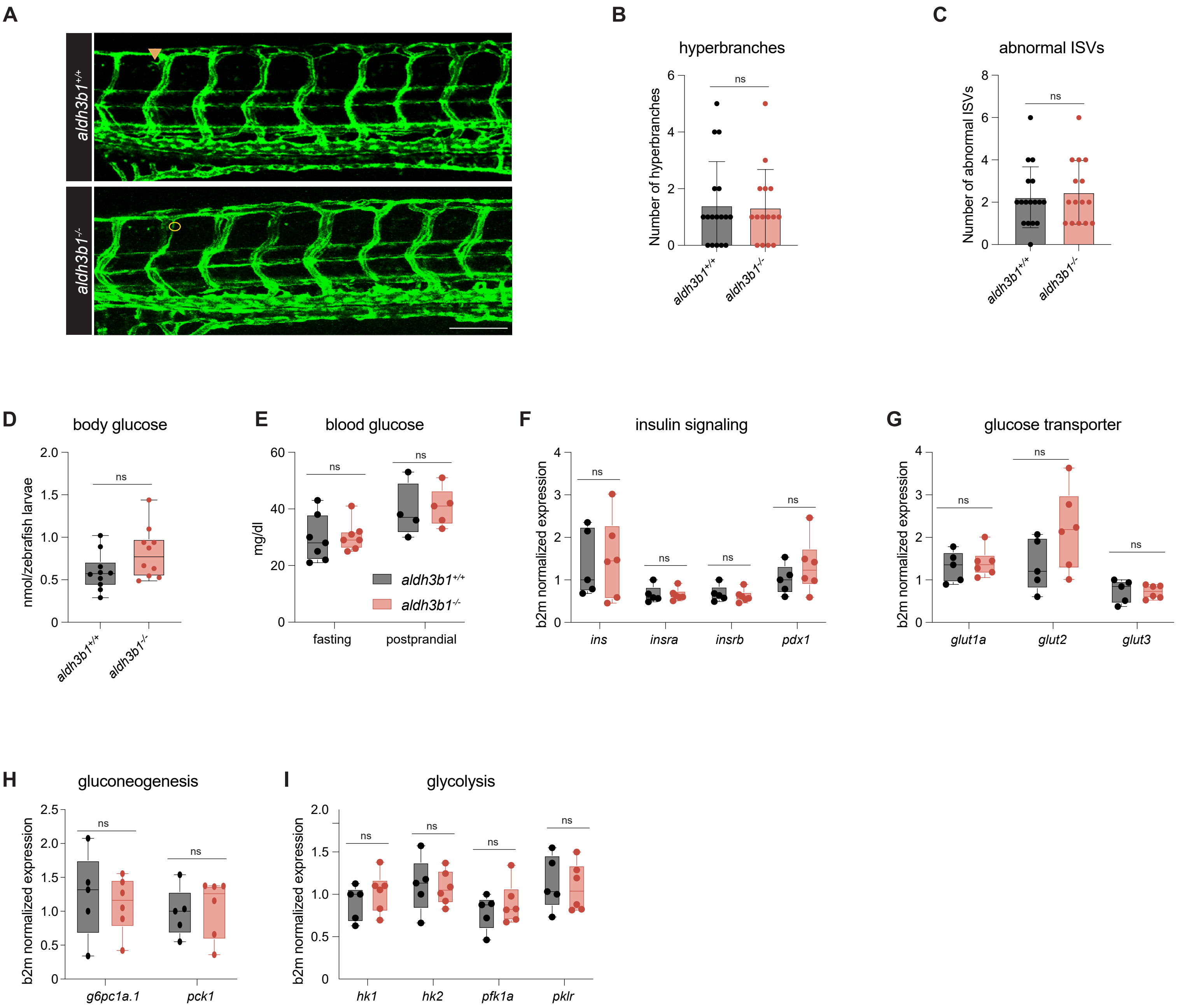


**Figure S1. Unaltered trunk vasculature and glucose metabolism in *aldh3b1$^{-/-}$* zebrafish**

(A) Representative confocal images of trunk vasculature in *aldh3b1$^{+/+}$* and *aldh3b1$^{-/-}$* larvae at 4dpf. Yellow arrow, hyperbranches; yellow circle, abnormal ISVs. White scale bar= 100 μm.

(B-C) Quantification of trunk vasculature revealed no significant differences in the hyperbranches and abnormal ISVs between *aldh3b1$^{+/+}$* and *aldh3b1$^{-/-}$* larvae (n=17/15).

(D) *aldh3b1* knockout did not alter the whole-body glucose level in 5dpf larvae (n=10/10).

(E) Fasting glucose levels (n=7/7) and 2-hour postprandial blood glucose levels (n=4/5) in adult zebrafish remain unaffected by *aldh3b1* knockout.

(F-I) Expression levels of key genes involved in glucose metabolism were not changed by *aldh3b1* knockout (*aldh3b1$^{+/+}$* = 6, *aldh3b1$^{-/-}$* = 6).

*ins, insulin; insra, insulin receptor a; insrb, insulin receptor b; pdx1, pancreatic and duodenal homeobox 1; glut, glucose transporter; g6pc1a.1, glucose-6-phosphatase catalytic subunit 1a; pck1, phosphoenolpyruvate carboxykinase 1; hk, hexokinase; pfkla, phosphofructokinase, liver a; pklr, pyruvate kinase L/R*; ISV, Intersegmental Vessel. Statistical analysis was performed using Student's t-test for B-I. The bars indicate mean ± SD values.

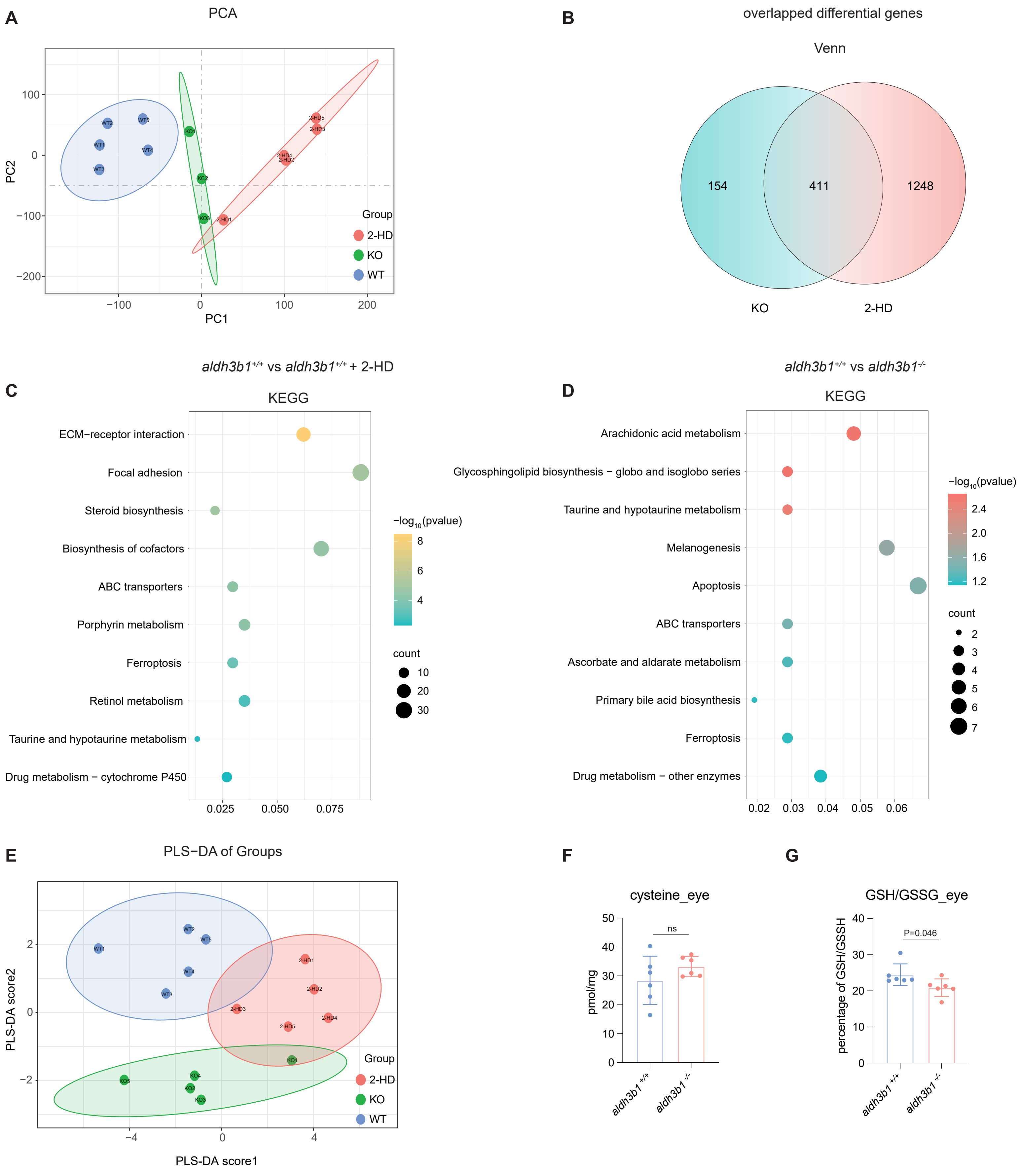


**Figure S2. Integrative analysis of RNA-seq and metabolomics in zebrafish models**

(A) PCA illustrates distinct clustering of sample groups of RNA-seq.

(B) Venn diagram representation of differentially expressed genes between *aldh3b1*$^{-/-}$ and 2-HD treated *aldh3b1*$^{+/+}$ larvae.

(C-D) KEGG enrichment analysis of differential genes identified the top 10 altered pathways in *aldh3b1*$^{-/-}$ and 2-HD treated *aldh3b1*$^{+/+}$ larvae.

(E) PLS-DA of metabolomics data displayed distinct metabolomic profiles.

(F-G) Analysis of ferroptosis-related metabolites in the eyes of *aldh3b1*$^{-/-}$ adults (n=6/6).

PCA, Principal Component Analysis; KEGG, Kyoto Encyclopedia of Genes and Genomes; PLS-DA, partial least squares-discriminant analysis.

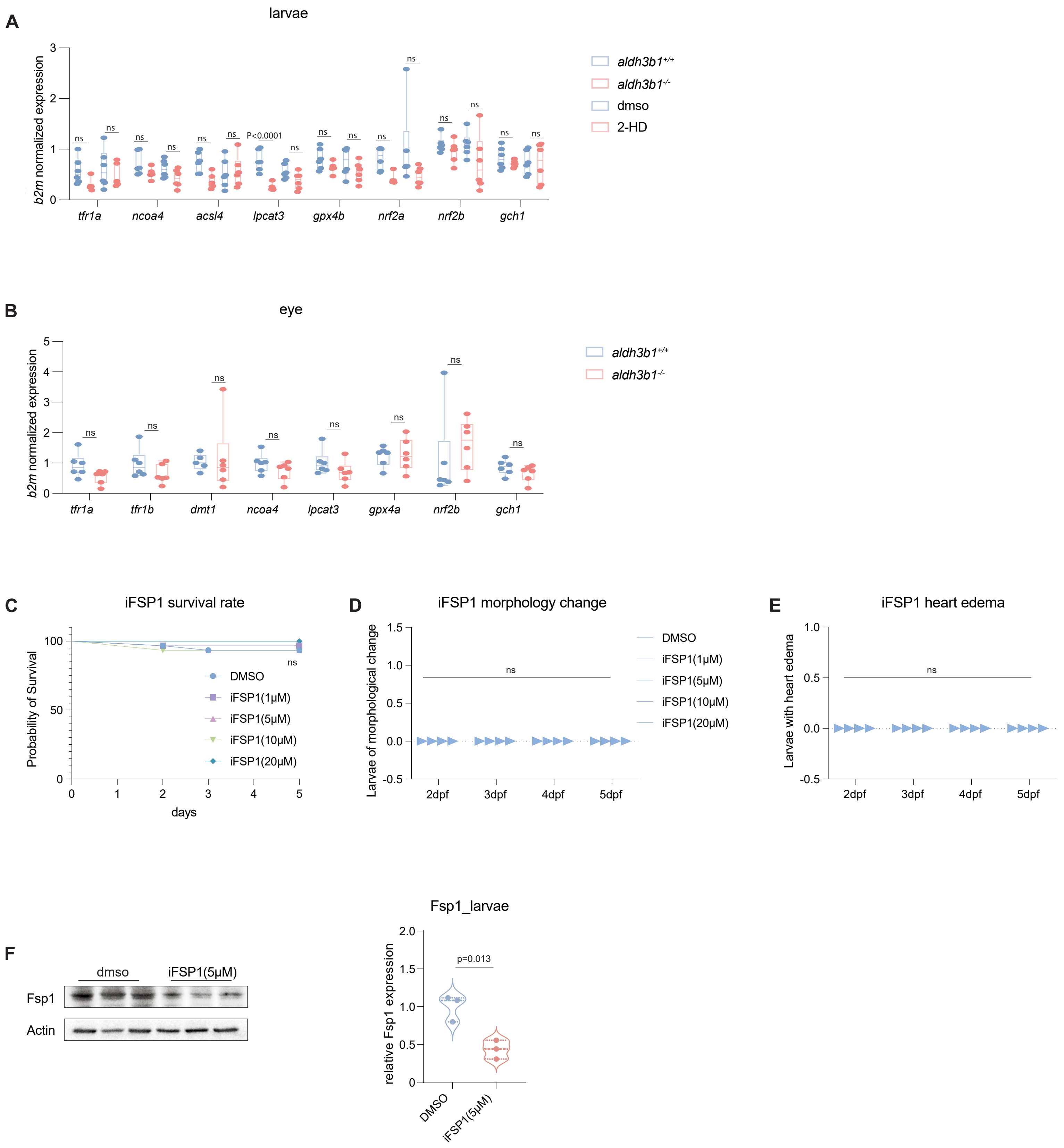


**Figure S3. Fsp1 downregulation-induced ferroptosis caused hyaloid vascular abnormalities**

(A-B) Quantitative mRNA expression analysis of ferroptosis related key genes in zebrafish larvae (n=6 for each group) and eyes (n=6/6).

(C-E) Exposure to 0 - 20 μmol iFSP1 did not affect survival rates (C), morphology (D) and heart development (E) in zebrafish larvae (n=3 for each concentration).

(F) Immunoblot analysis confirmed 5 μmol iFSP1 can decrease the Fsp1 protein level (n=3/3).

Statistical analysis was performed using one-way ANOVA for panel A, Student's t-test for B and F, Log-rank test for C, two-way ANOVA for D-E and. The bars indicate mean ± SD values.

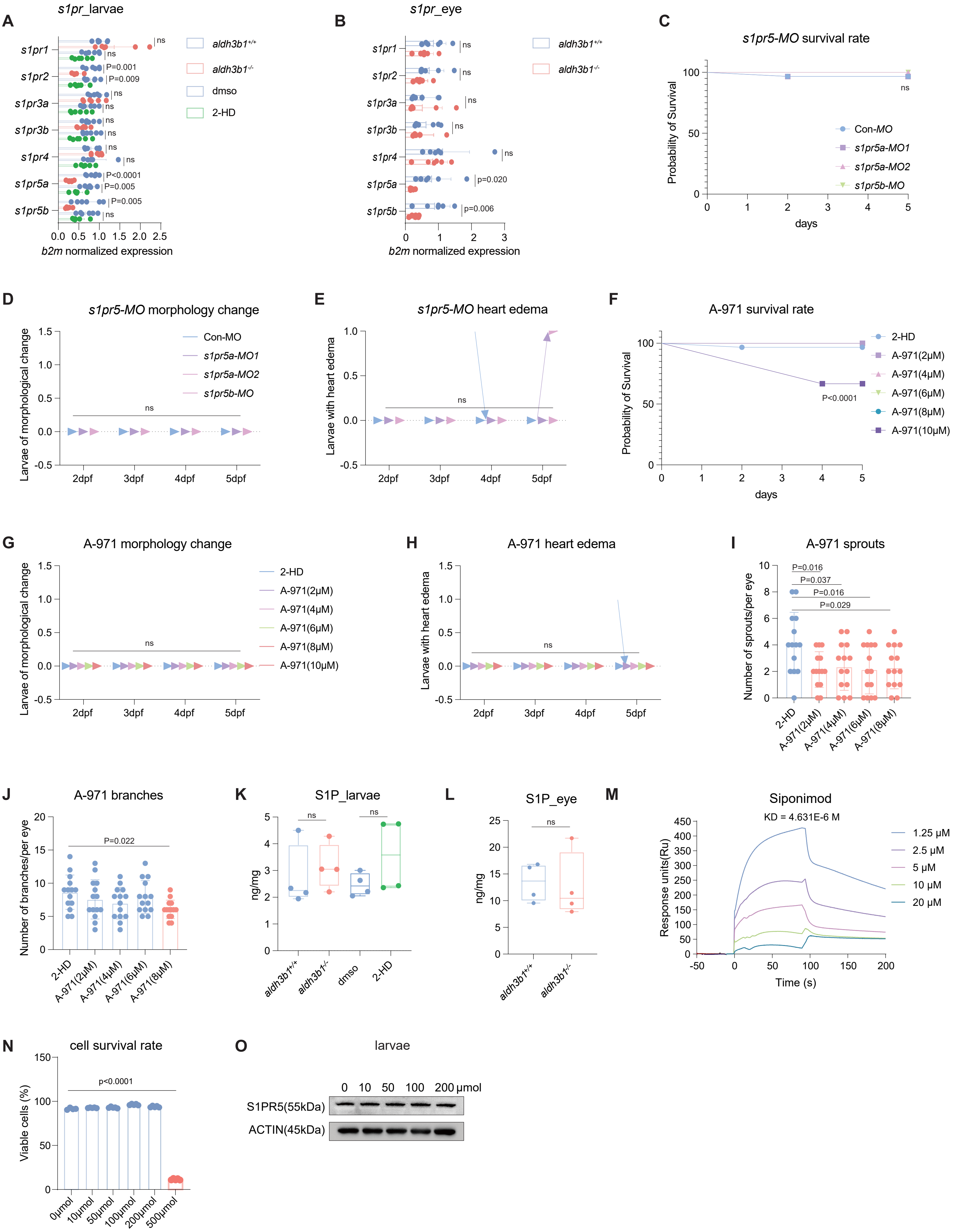

A
s1pr_larvae
s1pr1
s1pr2
s1pr3a
s1pr3b
s1pr4
s1pr5a
s1pr5b
ns
P=0.001
P=0.009
P<0.0001
P=0.005
P=0.005
0.0 0.5 1.0 1.5 2.0 2.5
b2m normalized expression
aldh3b1+/+
aldh3b1-/-
dmso
2-HD
B
s1pr_eye
p=0.020
p=0.006
0 1 2 3
b2m normalized expression
C
s1pr5-MO survival rate
Probability of Survival
days
Con-MO
s1pr5a-MO1
s1pr5a-MO2
s1pr5b-MO
D
s1pr5-MO morphology change
Larvae of morphological change
2dpf 3dpf 4dpf 5dpf
E
s1pr5-MO heart edema
Larvae with heart edema
F
A-971 survival rate
P<0.0001
2-HD
A-971(2μM)
A-971(4μM)
A-971(6μM)
A-971(8μM)
A-971(10μM)
G
A-971 morphology change
H
A-971 heart edema
I
A-971 sprouts
Number of sprouts/per eye
P=0.016
P=0.037
P=0.016
P=0.029
J
A-971 branches
Number of branches/per eye
P=0.022
K
S1P_larvae
ng/mg
L
S1P_eye
M
Siponimod
KD = 4.631E-6 M
Response units(Ru)
Time (s)
1.25 μM
2.5 μM
5 μM
10 μM
20 μM
N
cell survival rate
Viable cells (%)
p<0.0001
0μmol 10μmol 50μmol 100μmol 200μmol 500μmol
O
larvae
0 10 50 100 200 μmol
S1PR5(55kDa)
ACTIN(45kDa)

**Figure S4. S1pr5 regulates Fsp1 and hyaloid vasculature**

(A-B) Quantitative mRNA expression analysis of *s1p receptors* in zebrafish 5dpf larvae (A, n=6 for each group) and eyes (B, n=6/6).

(C-E) S1pr5 knockdown did not affect survival rates (C), morphology (D) and heart development (E) in zebrafish larvae (n=3 for each concentration).

(F) Treatment with 10 µmol A971 resulted in increased lethality of zebrafish larvae (n=3 for each concentration).

(G-H) Exposure to 0 - 10 µmol A971 did not affect morphology (G) and heart development (H) in zebrafish larvae (n=3 for each concentration).

(I-J) 8 µmol A971 showed the strongest rescue effect of 2-HD induced hyaloid vascular alterations (n=14 for each group).

(K-L) ELISA analysis revealed unaltered S1P level in larvae (K, n=4 for each group) and eye (L, n=4/4).

(M) Siponimod acts as positive control for SPR assay.

(N) Flow cytometry analysis revealed 500 µmol 2-HD significantly decreased cell survival rate in NK cells (n=5).

(O) 1 hour 2-HD incubation under indicated concentration did not significantly change total S1PR5 protein level.

Statistical analysis was performed using one-way ANOVA for panel A, I-K and N, Student's t-test for B and L, Log-rank test for C and F, and two-way ANOVA for D-E and G-H. The bars indicate mean ± SD values.

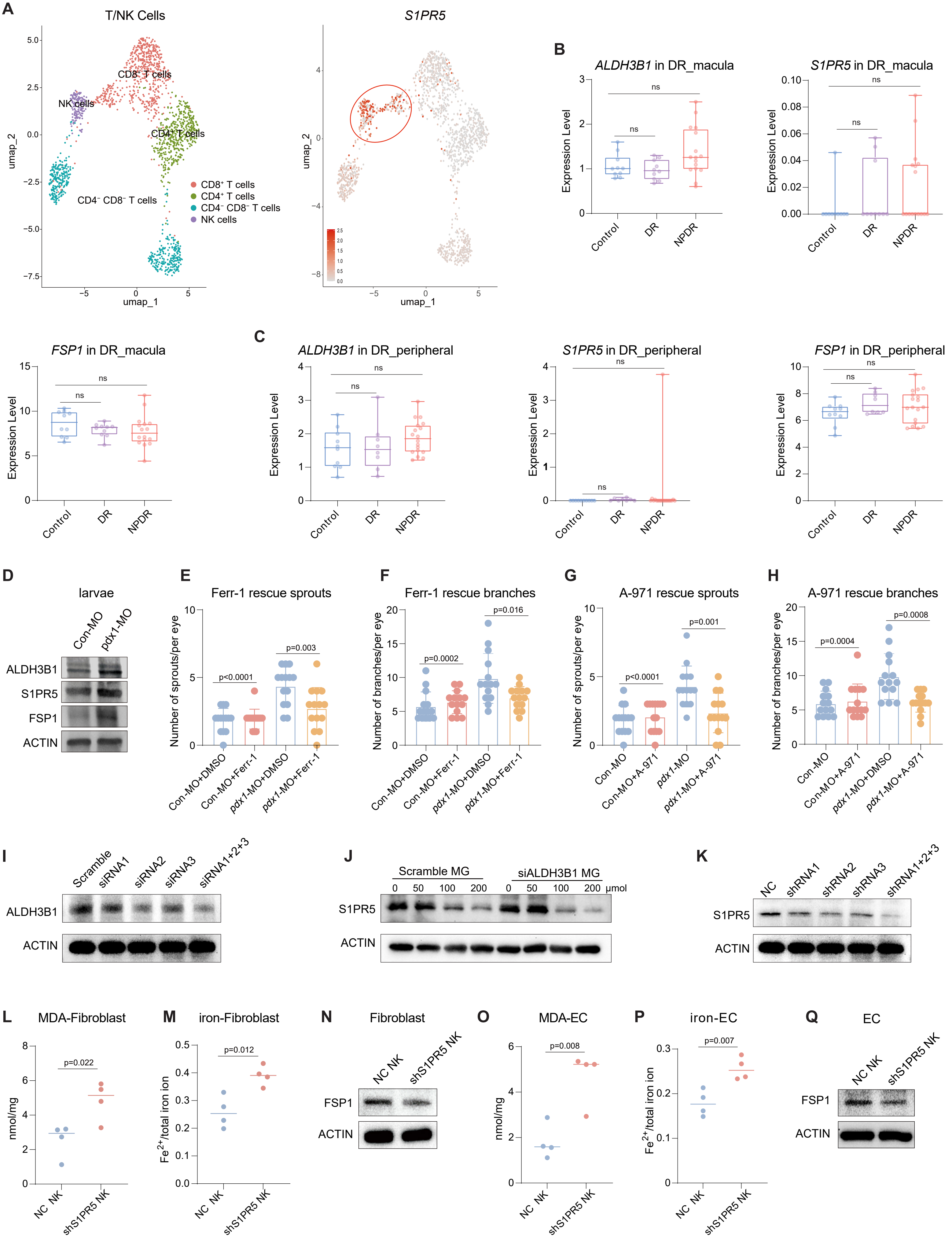
A
T/NK Cells
CD8⁺ T cells
NK cells
CD4⁺ T cells
CD4⁻ CD8⁻ T cells
umap_1
umap_2
S1PR5
B
ALDH3B1 in DR_macula
S1PR5 in DR_macula
Expression Level
ns
Control
DR
NPDR
FSP1 in DR_macula
C
ALDH3B1 in DR_peripheral
S1PR5 in DR_peripheral
FSP1 in DR_peripheral
D
larvae
Con-MO
pdx1-MO
ALDH3B1
S1PR5
FSP1
ACTIN
E
Ferr-1 rescue sprouts
Number of sprouts/per eye
p<0.0001
p=0.003
Con-MO+DMSO
Con-MO+Ferr-1
pdx1-MO+DMSO
pdx1-MO+Ferr-1
F
Ferr-1 rescue branches
Number of branches/per eye
p=0.0002
p=0.016
G
A-971 rescue sprouts
p<0.0001
p=0.001
Con-MO
Con-MO+A-971
pdx1-MO
pdx1-MO+A-971
H
A-971 rescue branches
p=0.0004
p=0.0008
pdx1-MO+DMSO
I
Scramble
siRNA1
siRNA2
siRNA3
siRNA1+2+3
ALDH3B1
ACTIN
J
Scramble MG
siALDH3B1 MG
0 50 100 200 0 50 100 200 μmol
S1PR5
ACTIN
K
NC
shRNA1
shRNA2
shRNA3
shRNA1+2+3
S1PR5
ACTIN
L
MDA-Fibroblast
nmol/mg
p=0.022
NC NK
shS1PR5 NK
M
iron-Fibroblast
Fe2+/total iron ion
p=0.012
N
Fibroblast
FSP1
ACTIN
O
MDA-EC
p=0.008
P
iron-EC
p=0.007
Q
EC

**Figure S5. S1PR5 is a novel target for human diabetic retinopathy**

(A) UMAP visualization of integrated single-cell RNA sequencing data revealed high *S1PR5* expression in NK cells.

(B–C) The expression of *ALDH3B1*, *S1PR5*, and *FSP1* remained unchanged in diabetic retinopathy (DR) and non-proliferative diabetic retinopathy (NPDR) samples in both the macular region (n = 10/10/16) and peripheral region (n = 10/8/18).

(D) Protein levels of Aldh1, S1pr5, and Fsp1 were increased in *pdx1*$^{-/-}$ zebrafish.

(E–H) The enhanced sprouting and branching of the hyaloid vasculature in *pdx1*$^{-/-}$ zebrafish were rescued by treatment with Ferrostatin-1 (E–F, n = 14 per group) or A971 (G–H, n = 14 per group).

(I) Efficient knockdown of ALDH3B1 in microglial cells was achieved using siRNA.

(J) Conditioned medium from siALDH3B1-treated microglia significantly reduced S1PR5 expression in NK cells.

(K) Efficient knockdown of S1PR5 in NK cells was achieved using shRNA.

(L–N) Fibroblasts cocultured with shS1PR5 NK cells exhibited elevated MDA levels (L) and increased iron accumulation (M), accompanied by decreased FSP1 expression (N) (n = 4).

(O–Q) Endothelial cells cocultured with shS1PR5 NK cells showed increased MDA levels (O) and iron accumulation (P), accompanied by decreased FSP1 expression (Q) (n = 4).

In panels J and L–Q, ALDH3B1 and S1PR5 was knocked down using a combination of siRNA1-3 and shRNA1-3 respectively. Statistical analysis was performed using one-way ANOVA for panels E–H and Student's t-test for panels L–M and O–P. Data are presented as mean ± SD.

## Supplementary table1 Oligonucleotide sequences used in this study

| Primer Name | Primer Sequence |
|---|---|
| 3b1_CRISPR_forward | GGAGGGCCAGAAGCAGG |
| 3b1_CRISPR_reverse | CCTGCTTCTGGCCCTCC |
| 3b1_genotyping_forward | ACAAATGCAGCAAATGCAACAA |
| 3b1_genotyping_reverse | TGTCAGCCTGAGCATATGTTTACC |
| b2m_qpcr_forward | GCAACGCTCTTTGTGAGGTG |
| b2m_qpcr_reverse | AACCACTGAACACGGACCTC |
| ins_qpcr_forward | GCCCAACAGGCTTCTTCTACAAC |
| ins_qpcr_reverse | GCAGATTTAGGAGGAAGGAAACCC |
| insra_qpcr_forward | AGAGGCCAGCGAGCTCTAC |
| insra_qpcr_reverse | CACTTGTGTGGGGGCTCT |
| insrb_qpcr_forward | GCCTCTGCGGATCACTACAT |
| insrb_qpcr_reverse | CTCCTGCGTGGTCTTGAAC |
| pdx1_qpcr_forward | ACACGCACGCATGGAAAGGACA |
| pdx1_qpcr_reverse | GCGGGCGCGAGATGTATTTGTT |
| glut1a_qpcr_forward | TGACCGGCCCATACGTTTTC |
| glut1a_qpcr_reverse | ATCATCTCGGTTATATTTATCTGCC |
| glut2_qpcr_forward | GCAGAAGAACCCTCACTC |
| glut2_qpcr_reverse | TCTCCGCCACAATAAACC |
| glut3_qpcr_forward | TCGTCAATGTCTTGGCTCTG |
| glut3_qpcr_reverse | CAACATACATTGGCGTGAGG |
| g6pc1a.1_qpcr_forward | GCTGCACCATACGAGATGGA |
| g6pc1a.1_qpcr_reverse | TCACCAAACAGCACCCACTT |
| pck1_qpcr_forward | GTGAACTGAACCGAGACCTG |
| pck1_qpcr_reverse | AGCACTTGAGAGCAAACGAT |
| pck1_qpcr_forward | GTGAACTGAACCGAGACCTG |
| pck1_qpcr_reverse | AGCACTTGAGAGCAAACGAT |
| hk1_qpcr_forward | ATGATAGCGGCACAGCTTCT |
| hk1_qpcr_reverse | GTTGGTGTCTCGTGCCAATC |
| hk2 _qpcr_forward | TGAGGTCAGTCTCGTCCAGT |
| hk2 _qpcr_reverse | TCTTAATCGACAGGCCACCG |
| pfkla_qpcr_forward | ACTGCCACTCCAGCGTTAAA |
| pfkla_qpcr_reverse | CAGAGCTGGAGTTCACCCTC |
| pklr_qpcr_forward | CAAAGGACACTTCCCTGTAGAG |
| pklr_qpcr_reverse | GGACAACGAGGACGATAACG |
| tfr1a_qpcr_forward | ATGAAGAGGTGGAGGCGAAC |
| tfr1a_qpcr_reverse | AGCGATTTCTGGTCTCACCG |
| tfr1b-qpcr-forward | CTGCCAGTCCTTTGTTGCAC |
| tfr1b-qpcr-reverse | TTTAGTTTGGTTGCGGCACG |
| DMT1 _qpcr_forward | TAATGAGCCCAGGAGCCGTA |
| DMT1 _qpcr_reverse | CTCTCTTACTGCTGTCTCGTGT |
| GPX4a_qpcr_forward | GAGGTTTACGCATCCTGGCT |
| GPX4a_qpcr_reverse | CTTGGATCCTGCAATGGGGA |
| GPX4b_qpcr_forward | CTGCAACCAGTTCGGAAAGC |
| GPX4b_qpcr_reverse | CCCAGTGTTCCTCTGCCTTT |
| FSP1_qpcr_forward | GGTGGCTGATCCTGAACTCC |
| FSP1_qpcr_reverse | TGCCCATCGCAATCAGCATA |
| LPCAT3_qpcr_forward | CCTCATTGTGTCCTCACGCT |
| LPCAT3_qpcr_reverse | TGGTTGTCCTGGAACATCGG |
| GCH1_qpcr_forward | GTGGTCGAAGCGACTCACAT |
| GCH1_qpcr_reverse | GGATCTTCGCGGAAAACACC |
| NRF2a_qpcr_forward | ACTCCAAACCTCCGTTCACC |
| NRF2a_qpcr_reverse | CGCCACTTTGTTCTTGCCTC |
| NRF2b_qpcr_forward | AGAAGCAGGGTTTCGGTGAG |
| NRF2b_qpcr_reverse | CAGAGGGCAGGCATGGTAAA |
| NCOA4_qpcr_forward | AGTTGCCGATTCGTAAGCGA |
| NCOA4_qpcr_reverse | GCAGGTCTCATTGGCTTTGC |
| S1PR1-qpcr-forward | TAGCCATCGCCATTGAACGA |
| S1PR1-qpcr-reverse | GTCGATGCAGTTCCATCCCA |
| S1PR2-qpcr-forward | CTACTTGCCGTCTGTTTGCG |
| S1PR2-qpcr-reverse | AGGCCAGGTTCCCAATGAAG |
| S1PR3a-qpcr-forward | ATGGATGACGAGCTTGAACC |
| S1PR3a-qpcr-reverse | TTCTTGGTCTTCCCCACTTG |
| S1PR3b-qpcr-forward | CCATTTGGAGGAACCACAAG |
| S1PR3b-qpcr-reverse | TCGGCTGGAGATAAATGGAG |
| S1PR4-qpcr-forward | TGAAGGCAGGATTGTGCGAT |
| S1PR4-qpcr-reverse | CGAAGGTGAAACTCCCTGCT |
| S1PR5a-qpcr-forward | GGGATGGACTGAAACCCGAG |
| S1PR5a-qpcr-reverse | GCTCCTTGGTACGGCTTCAT |
| S1PR5b-qpcr-forward | TGCAGCGCCACTACAACTAC |

| | |
|---|---|
| S1PR5b-qpcr-reverse | CACCACGTTCAGCATGTAGG |
| acsl4a-qpcr-forward | AGCAGGCACTATCACCGAAG |
| acsl4b-qpcr-reverse | TGTTTGCGTAAGCGCAGATG |
| Control-Mo | 5’-CCTCTTACCTCAGTTACAATTTATA-3' |
| s1pr5a-Mo#1 | 5' - AGCTTCCATTTCACTTCTGCTGACC - 3' |
| s1pr5a-Mo#2 | 5' - AAGCTTCCATTTCACTTCTGCTGAC- 3' |
| s1pr5b-Mo | 5' -CACACAGATGCTGCAGAACACTG- 3' |
| H-shS1PR5-1(656) | CCGGCTGCGTGCTCGCCTTCGTGGGCTCGAGCCCACGAAGGCGAGCACGCAGTTTTTT |
| H-shS1PR5-2(611) | CCGGTTGCTCCACTGTCTTGCCGCTCTCGAGAGCGGCAAGACAGTGGAGCAATTTTTT |
| H-shS1PR5-3(215) | CCGGCATCGTGCTAGAGAATCTAGCCTCGAGGCTAGATTCTCTAGCACGATGTTTTTT |
| ALDH3B1-human-99 | 5' - GCGAUGAGAGCGAUCGCUATT - 3' |
| ALDH3B1-human-416 | 5' - GGCAAGUUCUCCUUCGACATT - 3' |
| ALDH3B1-human-21 | 5' - GCCGCAUCAUCAACCAGAATT - 3' |
