# Supplementary material for: Sphingosine-1-Phosphate-derived 2-Hexadecenal is a central mediator of ocular neovascularization by inhibiting Sphingosine-1-Phosphate receptor 5
Source: Nat Commun. 2026 Apr 14;17:3488. doi: 10.1038/s41467-026-71792-3 (PMC13079744; doi:10.1038/s41467-026-71792-3)
Supplement: Supplementary file 2 — Reporting Summary [file 41467_2026_71792_MOESM2_ESM.pdf]

nature portfolio

Corresponding author(s): Jens Kroll

Last updated by author(s): Mar 01 2026

Reporting Summary

Nature Portfolio wishes to improve the reproducibility of the work that we publish. This form provides structure for consistency and transparency in reporting. For further information on Nature Portfolio policies, see our Editorial Policies and the Editorial Policy Checklist.

Statistics

For all statistical analyses, confirm that the following items are present in the figure legend, table legend, main text, or Methods section.

n/a	Confirmed	
☐	☒	The exact sample size (*n*) for each experimental group/condition, given as a discrete number and unit of measurement
☐	☒	A statement on whether measurements were taken from distinct samples or whether the same sample was measured repeatedly
☐	☒	The statistical test(s) used AND whether they are one- or two-sided<br>*Only common tests should be described solely by name; describe more complex techniques in the Methods section.*
☒	☐	A description of all covariates tested
☐	☒	A description of any assumptions or corrections, such as tests of normality and adjustment for multiple comparisons
☐	☒	A full description of the statistical parameters including central tendency (e.g. means) or other basic estimates (e.g. regression coefficient) AND variation (e.g. standard deviation) or associated estimates of uncertainty (e.g. confidence intervals)
☐	☒	For null hypothesis testing, the test statistic (e.g. *F*, *t*, *r*) with confidence intervals, effect sizes, degrees of freedom and *P* value noted<br>*Give P values as exact values whenever suitable.*
☒	☐	For Bayesian analysis, information on the choice of priors and Markov chain Monte Carlo settings
☒	☐	For hierarchical and complex designs, identification of the appropriate level for tests and full reporting of outcomes
☐	☒	Estimates of effect sizes (e.g. Cohen's *d*, Pearson's *r*), indicating how they were calculated

Our web collection on statistics for biologists contains articles on many of the points above.

Software and code

Policy information about availability of computer code

Data collection	QuantStudio 3 or QuantStudio 5 Real-Time PCR System (Thermo Fisher Scientific) was used to run real-time PCR; Vilber Fusion Solo S imaging system was used to collect data from immunoblotexperiment; Confocal microscope ((DM6000 B) with a Leica TCS SPS DS scanner was used to visualize and collect data of hyaloid vasculature; Plate reader (Tecan Infinite M200) was used to measure absorbance and fluorescence in the experiment of body glucose, lipid peroxidation assay, iron assay and ELISA. Glucometer (Freestyle Abbott) was used to measure blood glucose of adult fish.
Data analysis	These data are presented as mean±standard deviation (SD), as indicated in the figure legend. Statistical analysis of two groups was conducted using an unpaired Student's t-test, while one-way and two-way ANOVA were utilized for comparisons involving multiple groups with one or two variables, respectively, and the log-rank test was used for survival rate analysis. Correlation matrices were generated using Pearson's or Spearman's correlation coefficients. Statistical analysis was performed by Graph Pad Prism 8.0, R software (version 4.3.2) and Microsoft Excel (16.0). PyMOL (version 2.5.5) and Schrodinger (13.5) were used to perform molecular docking.

For manuscripts utilizing custom algorithms or software that are central to the research but not yet described in published literature, software must be made available to editors and reviewers. We strongly encourage code deposition in a community repository (e.g. GitHub). See the Nature Portfolio guidelines for submitting code & software for further information.

Data

Policy information about availability of data

All manuscripts must include a data availability statement. This statement should provide the following information, where applicable:

- Accession codes, unique identifiers, or web links for publicly available datasets
- A description of any restrictions on data availability
- For clinical datasets or third party data, please ensure that the statement adheres to our policy

The RNA-Seq datasets produced in this study are available at GEO (Gene Expression Omnibus, NIH) under the accession number: (https://www.ncbi.nlm.nih.gov/geo/query/acc.cgi?acc=GSE264377). The codes used for RNA-seq and single-cell RNA-seq analyses are available on GitHub at https://github.com/hasionwojoe/R-code and archived at Zenodo (DOI: 10.5281/zenodo.18772489). Other generated data supporting this paper are presented within the Supplementary Materials. Source data are provided with this paper.

Research involving human participants, their data, or biological material

Policy information about studies with human participants or human data. See also policy information about sex, gender (identity/presentation), and sexual orientation and race, ethnicity and racism.

Reporting on sex and gender	Cells were obtained from a single healthy donor. The sex of the donor was male. As all experiments were performed using cells from the same donor, sex was not a biological variable in this study.
Reporting on race, ethnicity, or other socially relevant groupings	Race and ethnicity were not collected, as they were not relevant to the aims of this study and the experiments were performed using primary cells from a single healthy donor.
Population characteristics	Primary human NK cells were obtained from one healthy adult donor. No additional population characteristics were collected because the study did not involve a human cohort.
Recruitment	The donor was not recruited for this study. Peripheral blood was obtained from a healthy volunteer under institutional approval and informed consent.
Ethics oversight	Peripheral blood samples were obtained from donors in the Department of Vascular Surgery, Renji Hospital, in accordance with a study protocol approved by the Institutional Review Board of Shanghai Jiao Tong University School of Medicine, Renji Hospital (Approval No. KY2024–170-C). All procedures involving human participants adhered to the ethical principles of the Declaration of Helsinki. Written informed consent was obtained from all participants or their legal guardians prior to specimen collection.

Note that full information on the approval of the study protocol must also be provided in the manuscript.

Field-specific reporting

Please select the one below that is the best fit for your research. If you are not sure, read the appropriate sections before making your selection.

☒ Life sciences ☐ Behavioural & social sciences ☐ Ecological, evolutionary & environmental sciences

For a reference copy of the document with all sections, see nature.com/documents/nr-reporting-summary-flat.pdf

Life sciences study design

All studies must disclose on these points even when the disclosure is negative.

Sample size	In in larvae studies, greater than 60 larvae were analyzed in each experiment group to permit statistical comparisons. In adult fish studies, at least 4 fish were included in each group. For all other studies, no statistical method was used to predetermine sample size.
Data exclusions	No data was excluded from the analyses in thisstudy.
Replication	Experiments were conducted with technical and biological replicates as indicated in the Figure Legends. Reported findings were successfully replicated.
Randomization	For the larvae experiments, the larvae were evenly distributed into culture dishes and subjected to different treatments; randomization was not required. For the adult fish experiments, we used adults from the same batch and parental lineage.
Blinding	Blinding was not performed as no human or patient information was analyzed in this study. Blinding was not necessary as all samples in the same experiment were collected and analyzedunder the same conditions.

Reporting for specific materials, systems and methods

We require information from authors about some types of materials, experimental systems and methods used in many studies. Here, indicate whether each material, system or method listed is relevant to your study. If you are not sure if a list item applies to your research, read the appropriate section before selecting a response.

Materials & experimental systems

n/a	Involved in the study
☐	☒ Antibodies
☐	☒ Eukaryotic cell lines
☒	☐ Palaeontology and archaeology
☐	☒ Animals and other organisms
☒	☐ Clinical data
☒	☐ Dual use research of concern
☒	☐ Plants

Methods

n/a	Involved in the study
☒	☐ ChIP-seq
☐	☒ Flow cytometry
☒	☐ MRI-based neuroimaging

Antibodies

Antibodies used	primary antibodies (anti-Actin(1:1000), Santa Cruz Biotechnology, sc-47778; anti-ALDH3B1(1:500), abcam, ab236673; anti-AMID(1:1000), Santa Cruz Biotechnology, sc-377120; anti-GPX4(1:1000), Santa Cruz Biotechnology, sc-166570; anti-S1PR5(1:500), Proteintech, 13874-1-AP), followed by secondary HRP-conjugated antibodies1:1000(rabbit anti mouse, DAKO, P0260; Goat anti-Rabbit, Dako, P0448).
Validation	All the antibodies were well-recognized clones in the field and validated by the manufacturers.

Eukaryotic cell lines

Policy information about cell lines and Sex and Gender in Research

Cell line source(s)	Human fibroblasts (HEF, SCSP -106) were purchased from National Collection of Authenticated Cell Cultures, China. Human microglia was donated by Dr. Yan Li. Human umbilical vein endothelial cells (HUVECs; DFSC-EC-01) was purchase fromZQXZbio, China.
Authentication	Cellauthentication was conducted using short tandem repeat (STR) analysis (Genetic Testing Biotechnology Corporation,liangsu, China).
Mycoplasma contamination	All cell lines were cultured with a low concentration of mycoplasma removal agent (0.5 ug/ml, Beyotime) according to themanufacturer's instructions. The cell lines were subsequently tested and confirmed to be negative for mycoplasmacontamination.
Commonly misidentified lines (See ICLAC register)	No commonly misidentified cell line was used.

Animals and other research organisms

Policy information about studies involving animals; ARRIVE guidelines recommended for reporting animal research, and Sex and Gender in Research

Laboratory animals	The zebrafish line Tg(fli1:EGFP) were used in this study, from larvae to 15 months old.
Wild animals	The study did not involved wild animals.
Reporting on sex	To avoid additional variables from mixed sex populations, all female or all male fish were used in this study, as indicated in the Methods.
Field-collected samples	The study did not involve samples collected in the field.
Ethics oversight	All the zebrafish experimental protocols were approved by the local government authority Regierungsprasidum-Karlsruhe and by Medical Faculty Mannheim (license no: G-98/15 and I-19/02).

Note that full information on the approval of the study protocol must also be provided in the manuscript.

Plants

Seed stocks: /

Novel plant genotypes: /

Authentication: /

Flow Cytometry

Plots

Confirm that:

- [ ] The axis labels state the marker and fluorochrome used (e.g. CD4-FITC).
- [ ] The axis scales are clearly visible. Include numbers along axes only for bottom left plot of group (a 'group' is an analysis of identical markers).
- [ ] All plots are contour plots with outliers or pseudocolor plots.
- [x] A numerical value for number of cells or percentage (with statistics) is provided.

Methodology

Sample preparation	Cells were harvested and washed in PBS twice, then subsequently stained as described in the methods section.
Instrument	BD FACSAriaTM Ill and BD FACSCelestaTM
Software	BD FACS Diva
Cell population abundance	NK cells were defined as viable CD3+ CD56+ lymphocytes. The final sorted population purity consistently exceeded 95%.
Gating strategy	Initial gating was performed on FSC/SSC to select lymphocytes, followed by exclusion of dead cells using LIVE/DEAD viability dye. NK cells were identified as CD3+CD56+ populations.

- [x] Tick this box to confirm that a figure exemplifying the gating strategy is provided in the Supplementary Information.
