# Supplementary material for: Sphingosine-1-Phosphate-derived 2-Hexadecenal is a central mediator of ocular neovascularization by inhibiting Sphingosine-1-Phosphate receptor 5
Source: Nat Commun. 2026 Apr 14;17:3488. doi: 10.1038/s41467-026-71792-3 (PMC13079744; doi:10.1038/s41467-026-71792-3)

Figure 1H

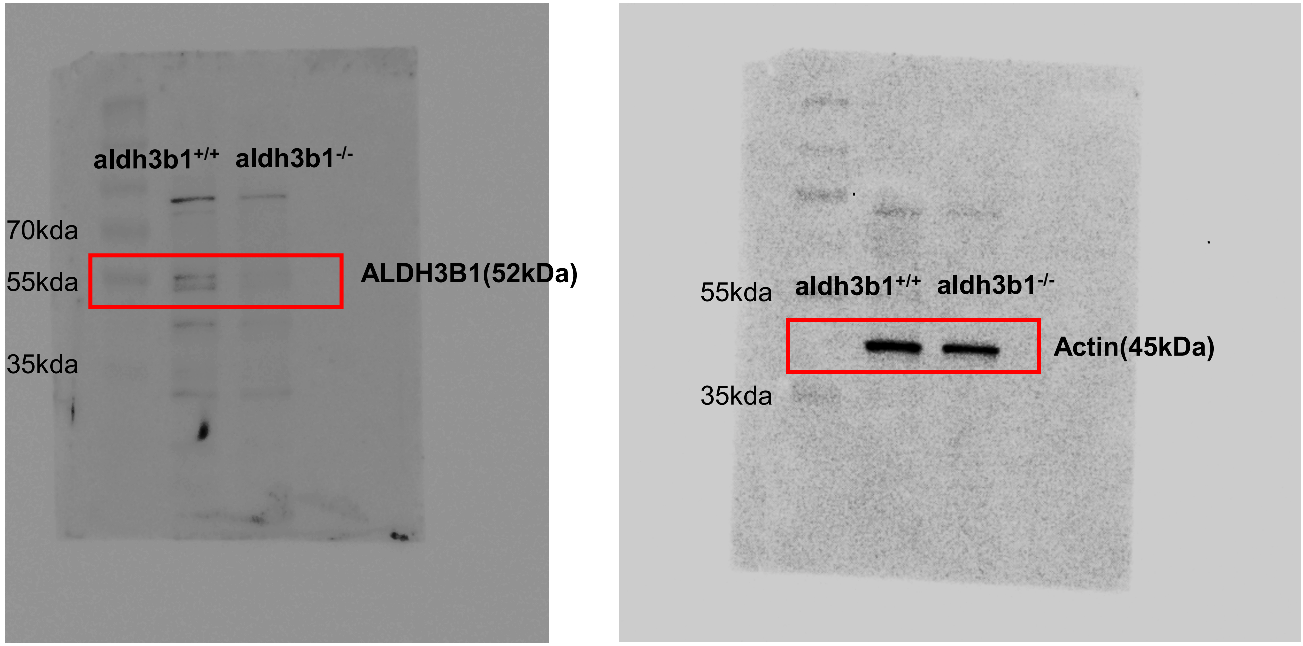
aldh3b1$^{+/+}$ aldh3b1$^{-/-}$
70kda
55kda
ALDH3B1(52kDa)
35kda
55kda
aldh3b1$^{+/+}$ aldh3b1$^{-/-}$
Actin(45kDa)
35kda

Figure 5G

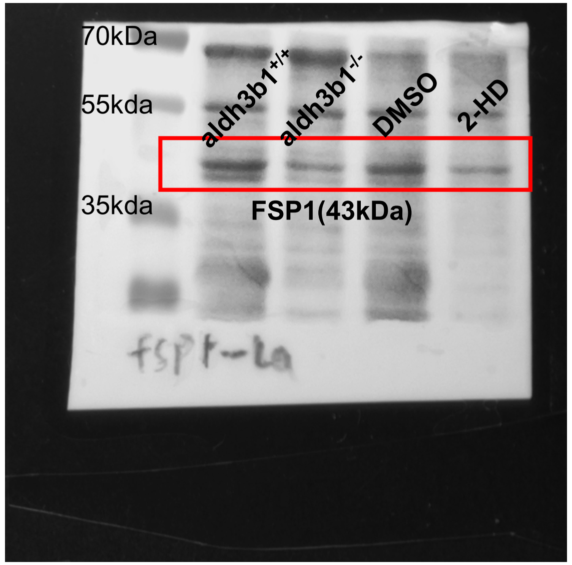
70kDa
55kda
35kda
aldh3b1+/+
aldh3b1-/-
DMSO
2-HD
FSP1(43kDa)

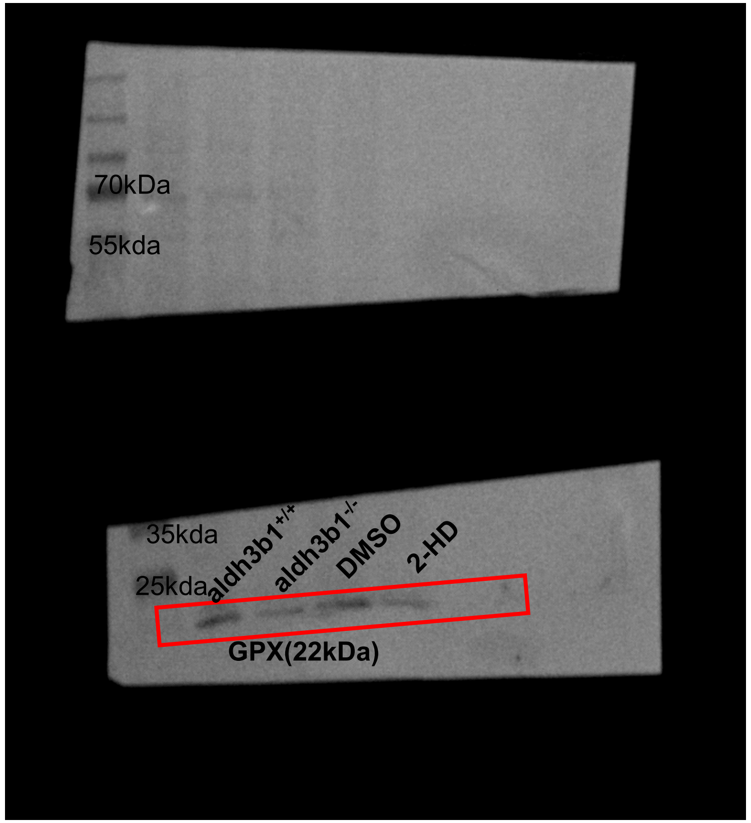
70kDa
55kda
35kda
25kda
aldh3b1+/+
aldh3b1-/-
DMSO
2-HD
GPX(22kDa)

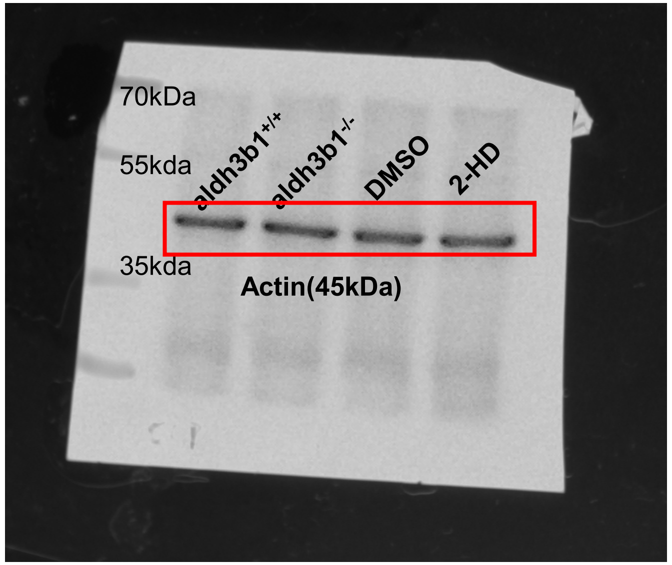
70kDa
55kda
35kda
aldh3b1+/+
aldh3b1-/-
DMSO
2-HD
Actin(45kDa)

Figure 5K

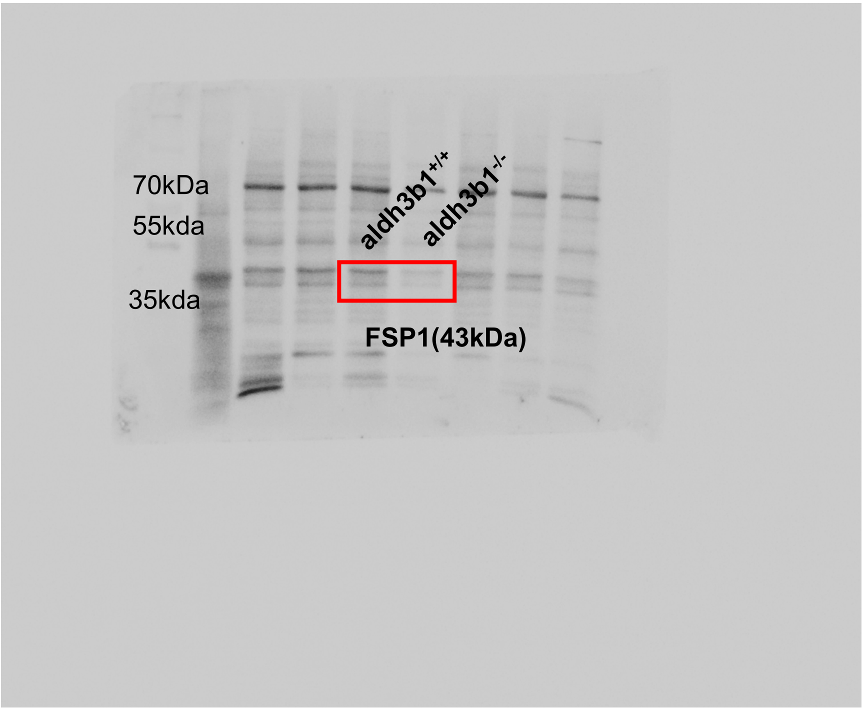
70kDa
55kda
aldh3b1$^{+/+}$
aldh3b1$^{-/-}$
35kda
FSP1(43kDa)

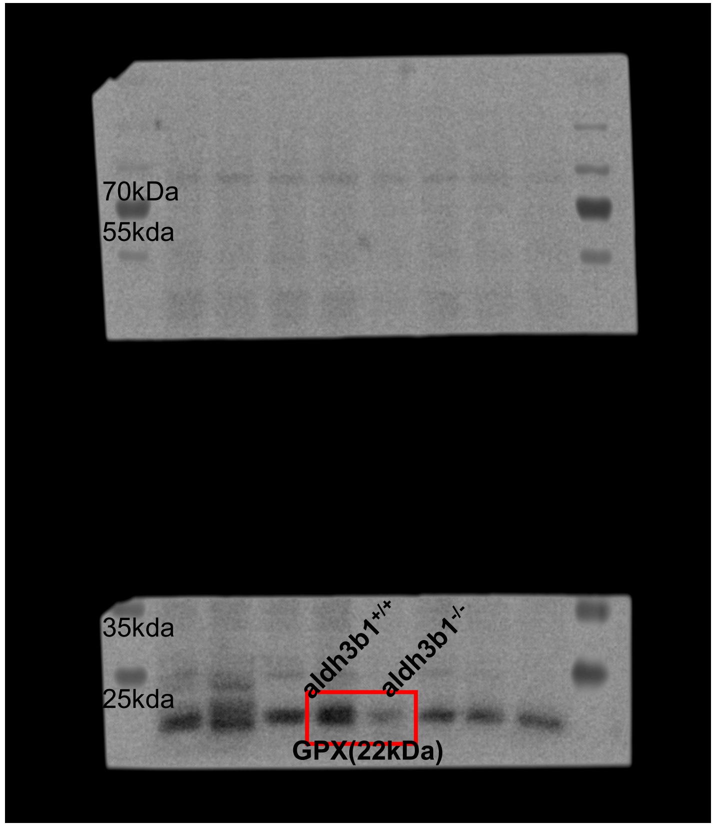
70kDa
55kda
35kda
aldh3b1$^{+/+}$
aldh3b1$^{-/-}$
25kda
GPX(22kDa)

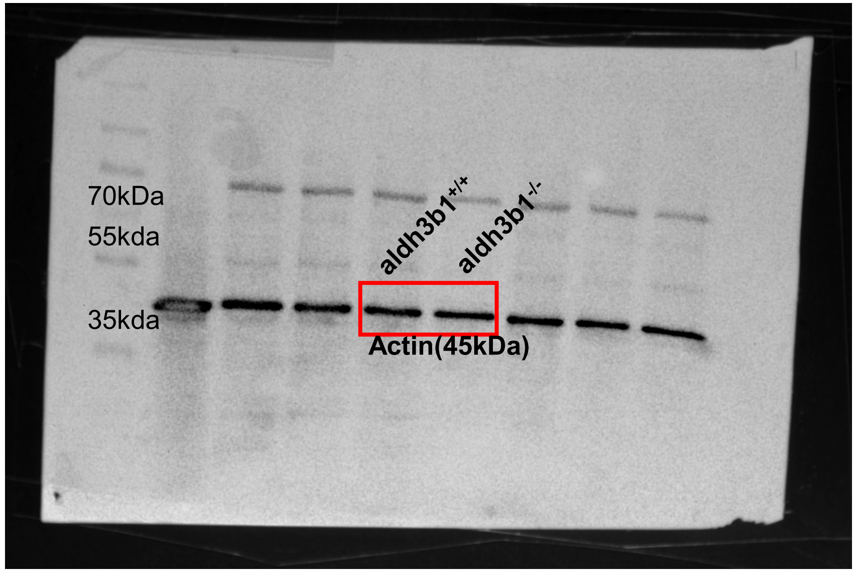
70kDa
aldh3b1$^{+/+}$
aldh3b1$^{-/-}$
55kda
35kda
Actin(45kDa)

Figure 6B

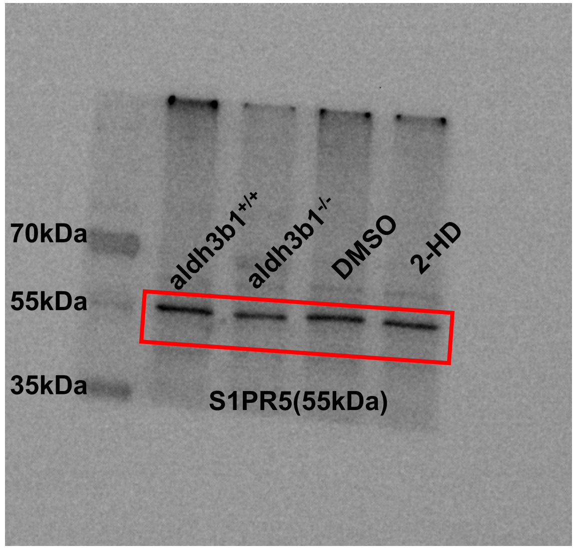

70kDa
55kDa
35kDa
aldh3b1+/+
aldh3b1-/-
DMSO
2-HD
S1PR5(55kDa)

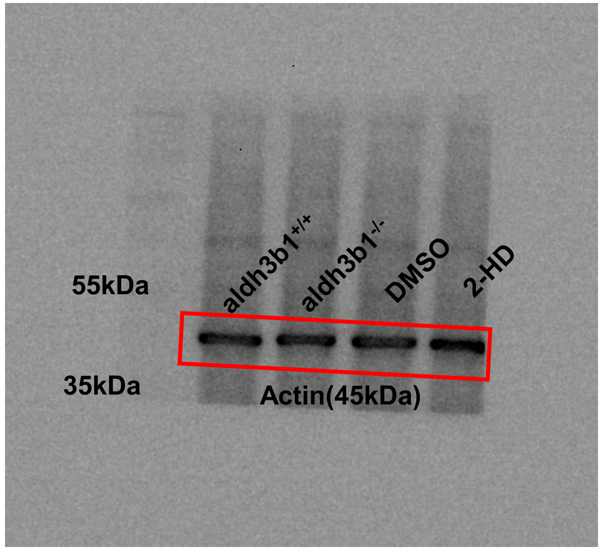

55kDa
35kDa
aldh3b1+/+
aldh3b1-/-
DMSO
2-HD
Actin(45kDa)

Figure 6C

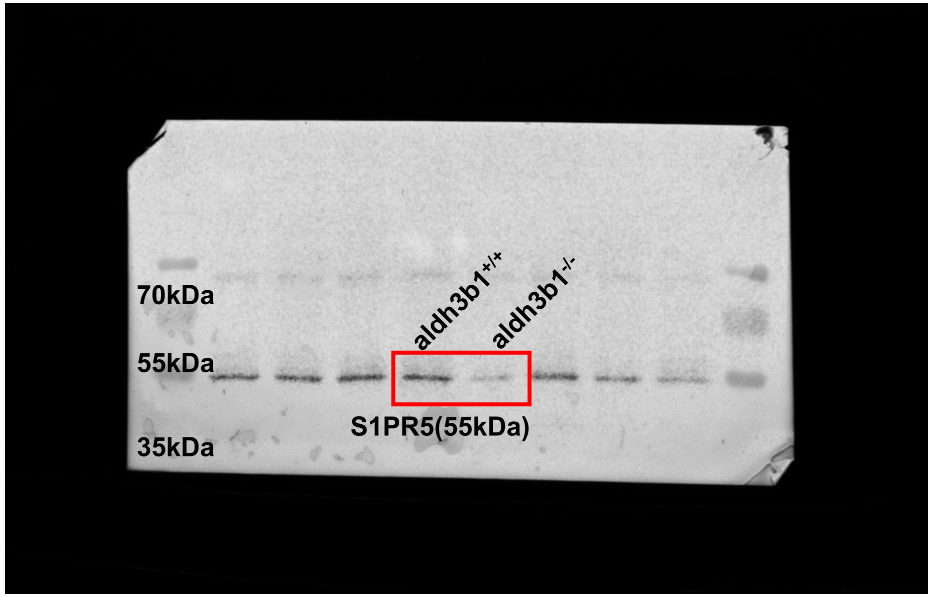
70kDa
55kDa
35kDa
aldh3b1+/+
aldh3b1-/-
S1PR5(55kDa)

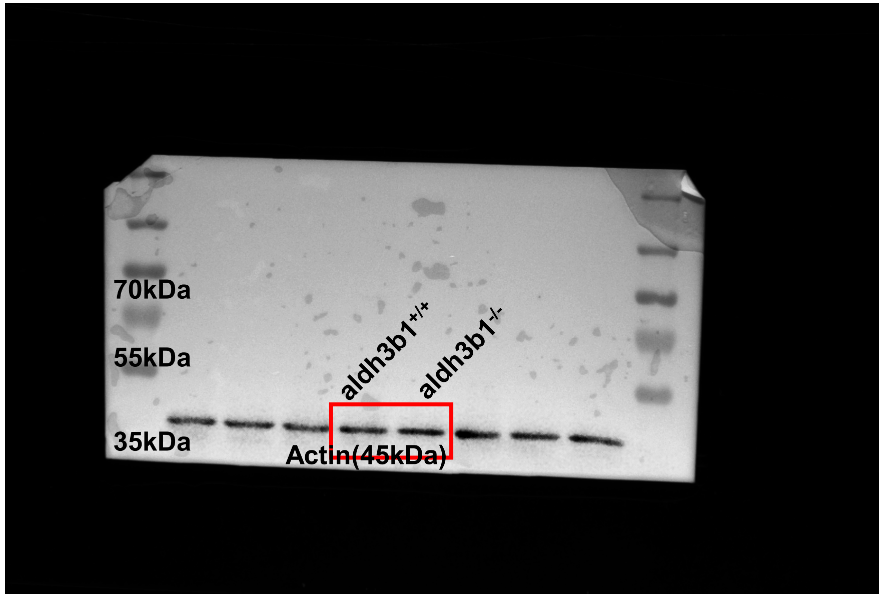
70kDa
55kDa
35kDa
aldh3b1+/+
aldh3b1-/-
Actin(45kDa)

Figure 6F

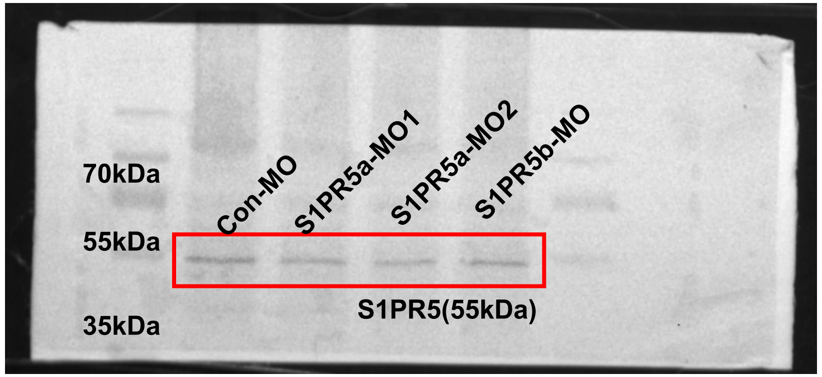
70kDa
55kDa
35kDa
Con-MO
S1PR5a-MO1
S1PR5a-MO2
S1PR5b-MO
S1PR5(55kDa)

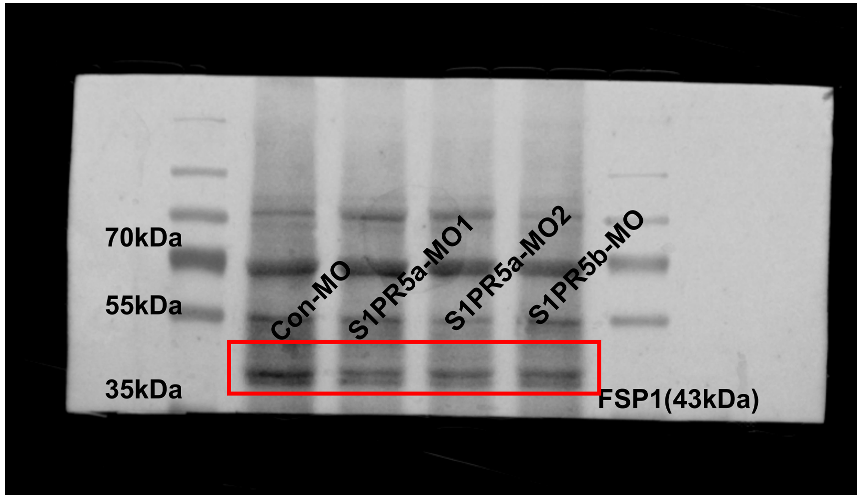
70kDa
55kDa
35kDa
Con-MO
S1PR5a-MO1
S1PR5a-MO2
S1PR5b-MO
FSP1(43kDa)

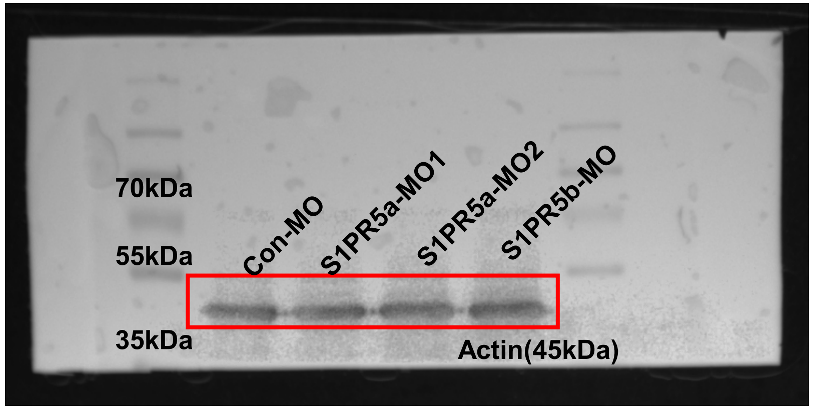
70kDa
55kDa
35kDa
Con-MO
S1PR5a-MO1
S1PR5a-MO2
S1PR5b-MO
Actin(45kDa)

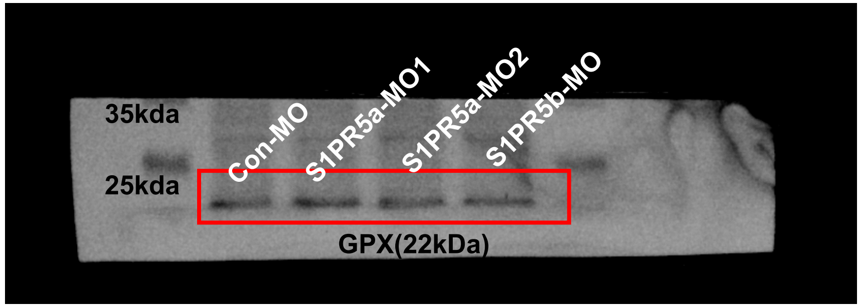
35kda
25kda
Con-MO
S1PR5a-MO1
S1PR5a-MO2
S1PR5b-MO
GPX(22kDa)

Supplementary Figure 3F

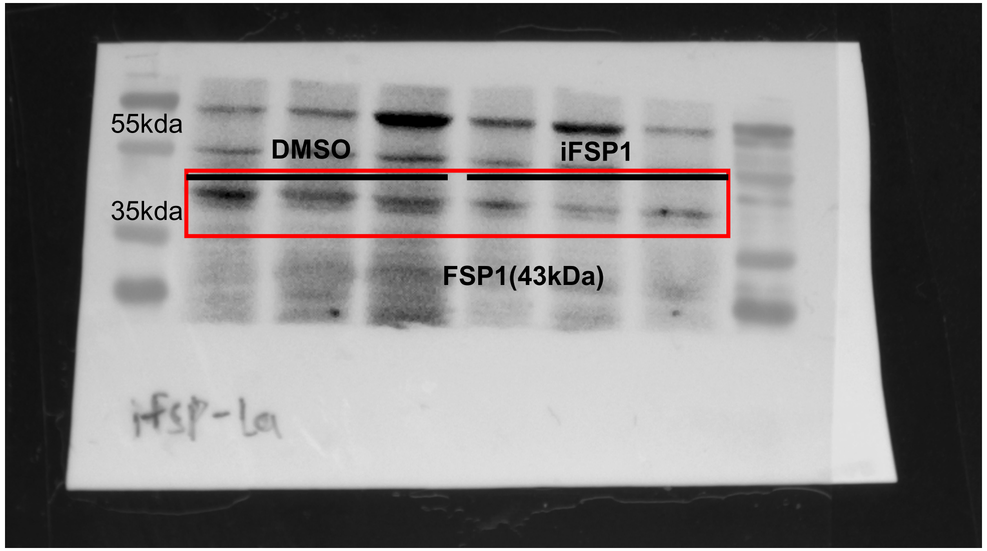

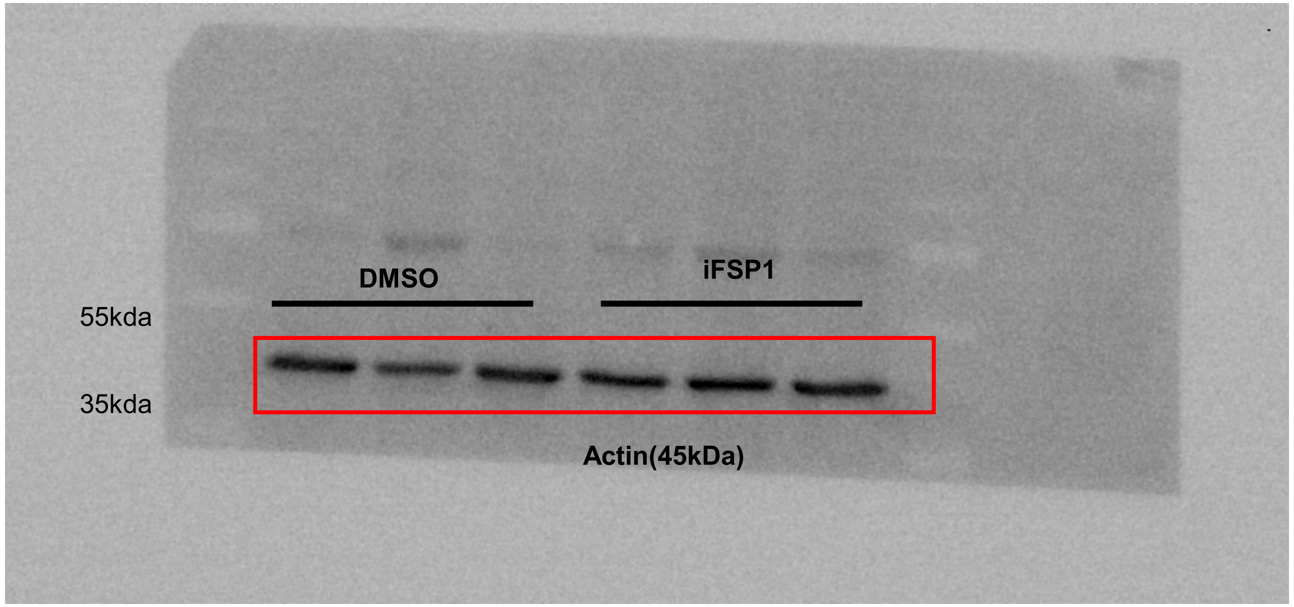

Supplementary Figure 4O

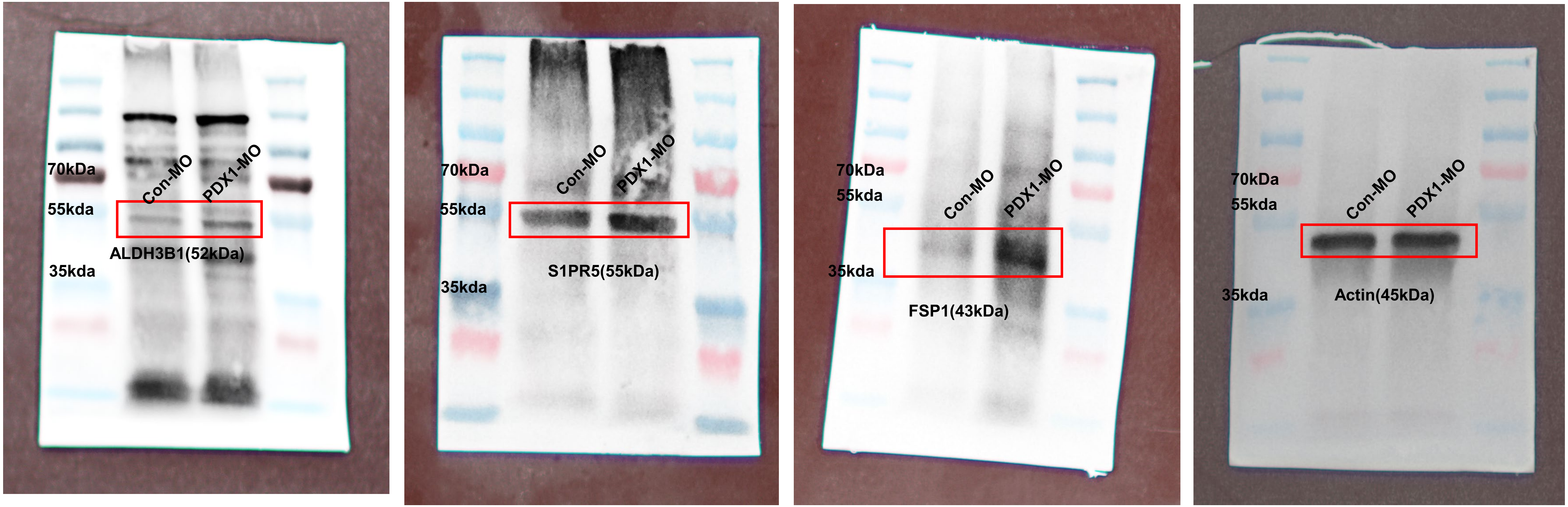

Supplementary Figure 6D

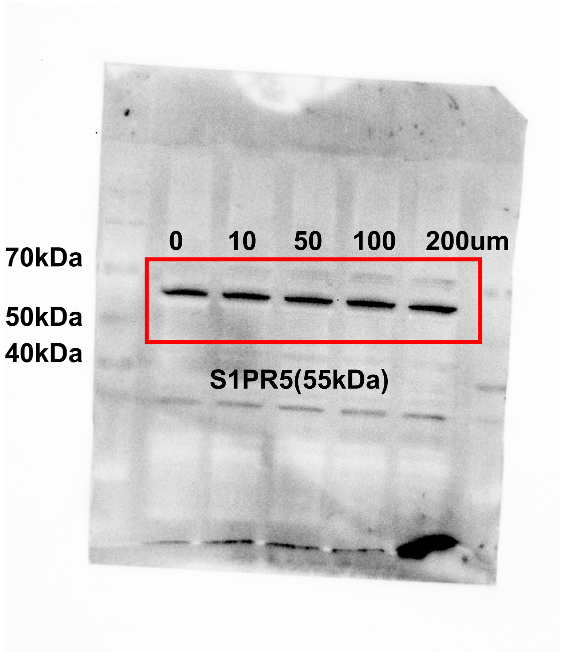

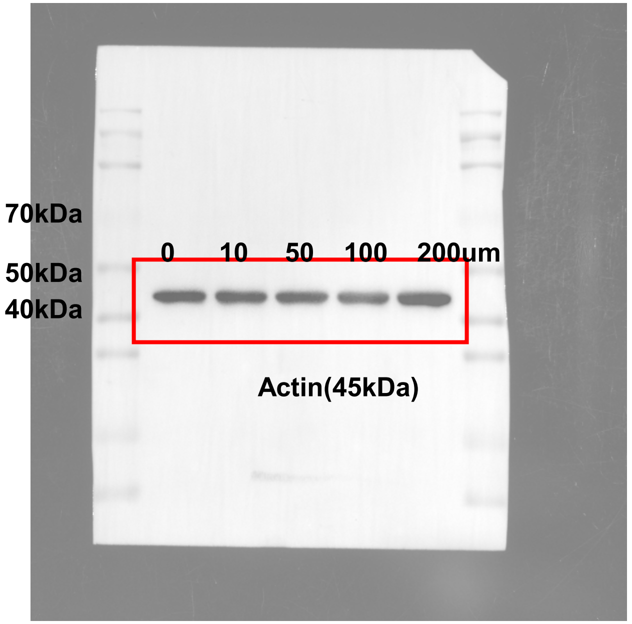

Supplementary Figure 6I

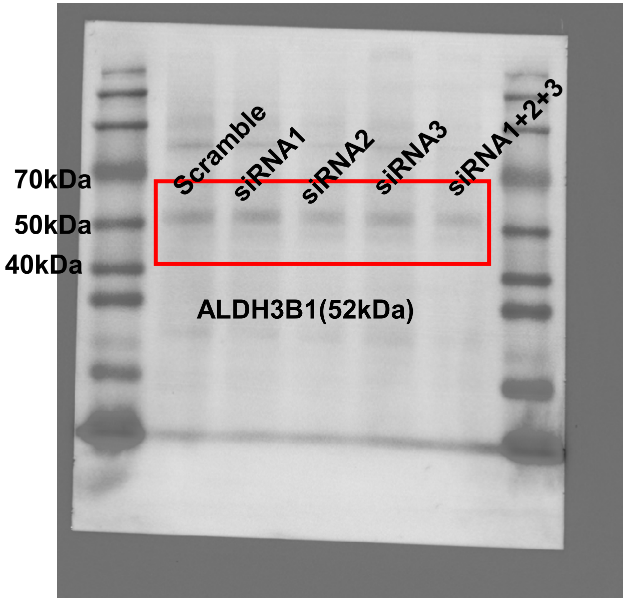

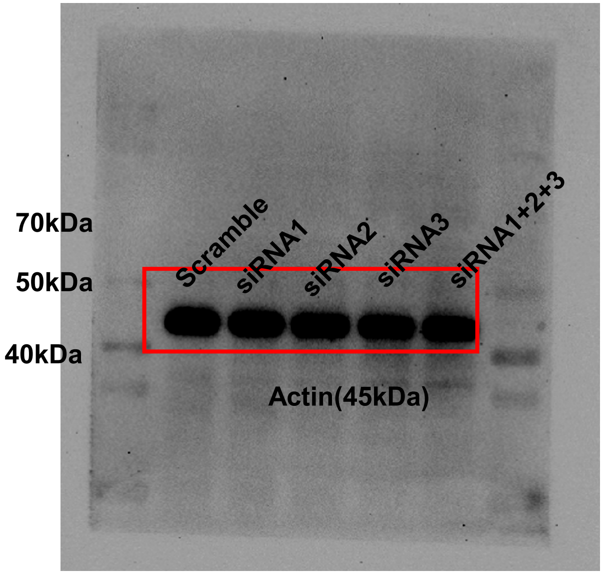

Supplementary Figure 6J

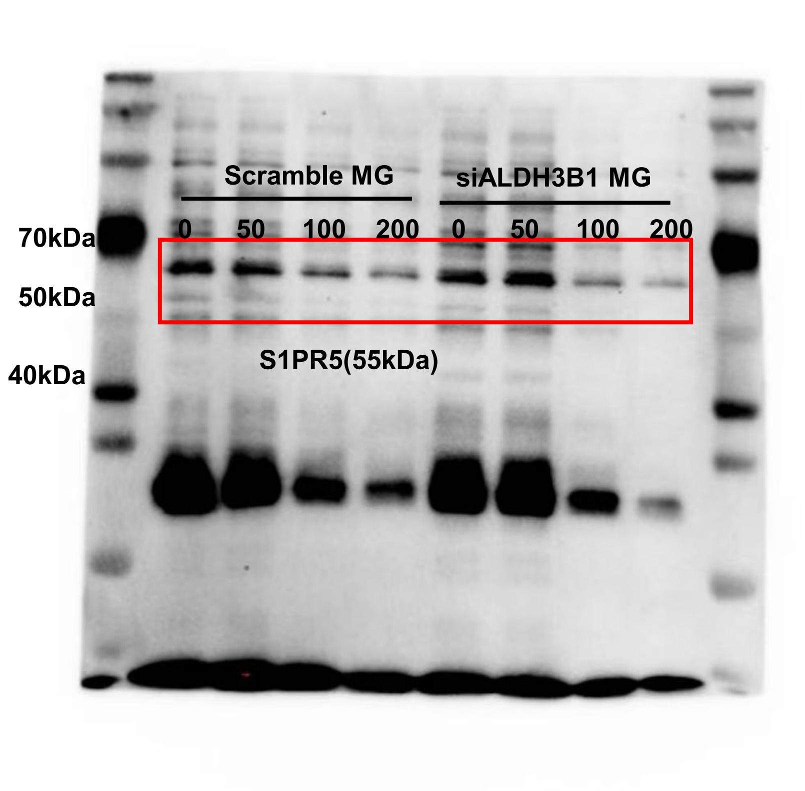

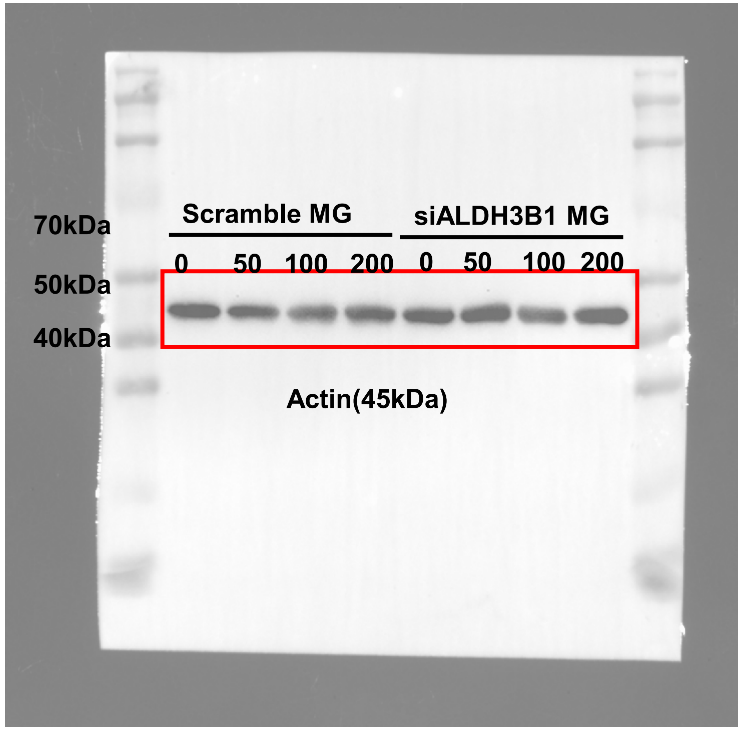

Supplementary Figure 6K

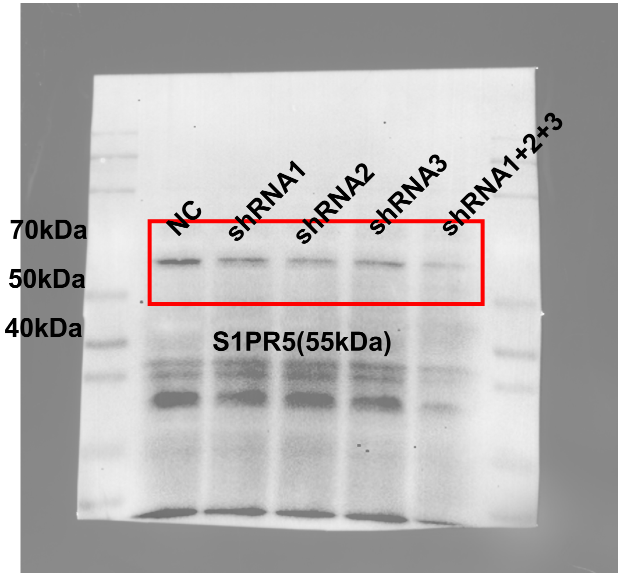

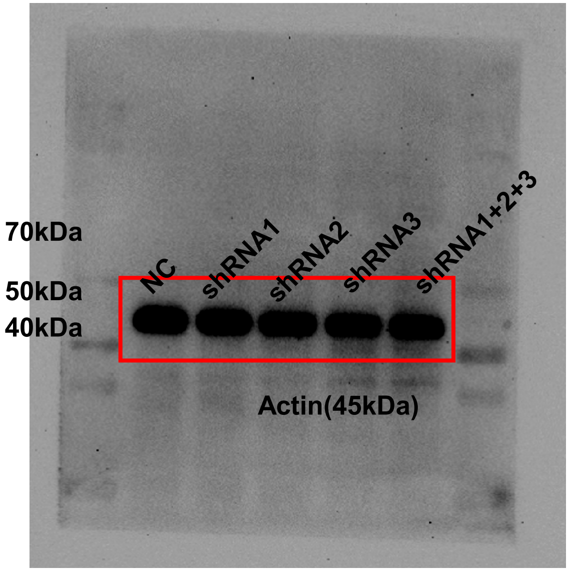

Supplementary Figure 6N

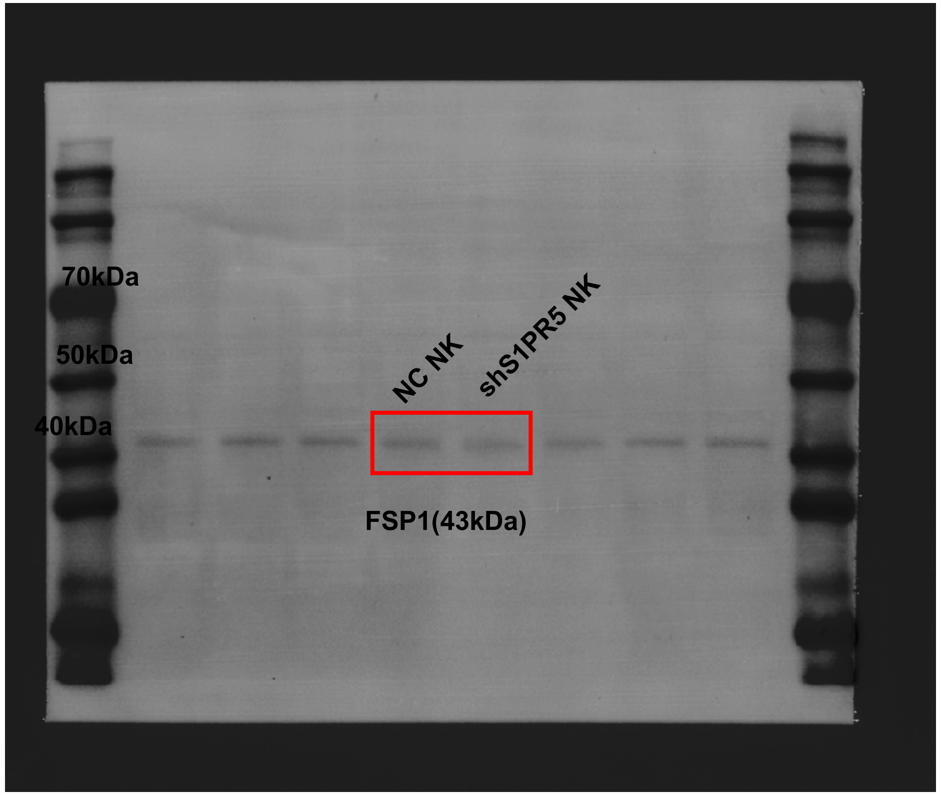

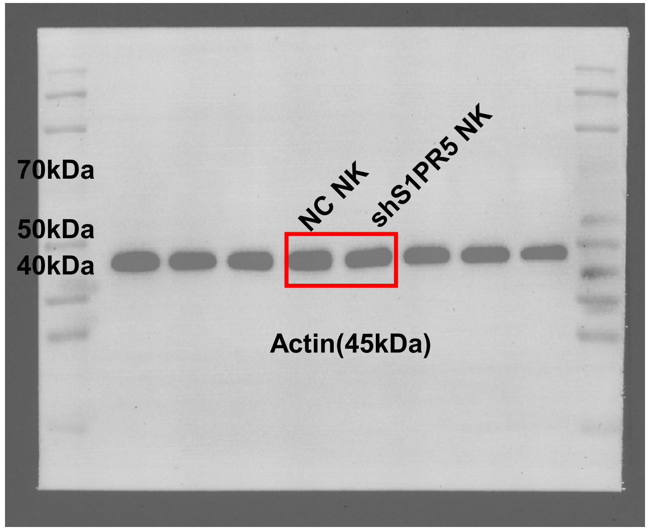

Supplementary Figure 6Q

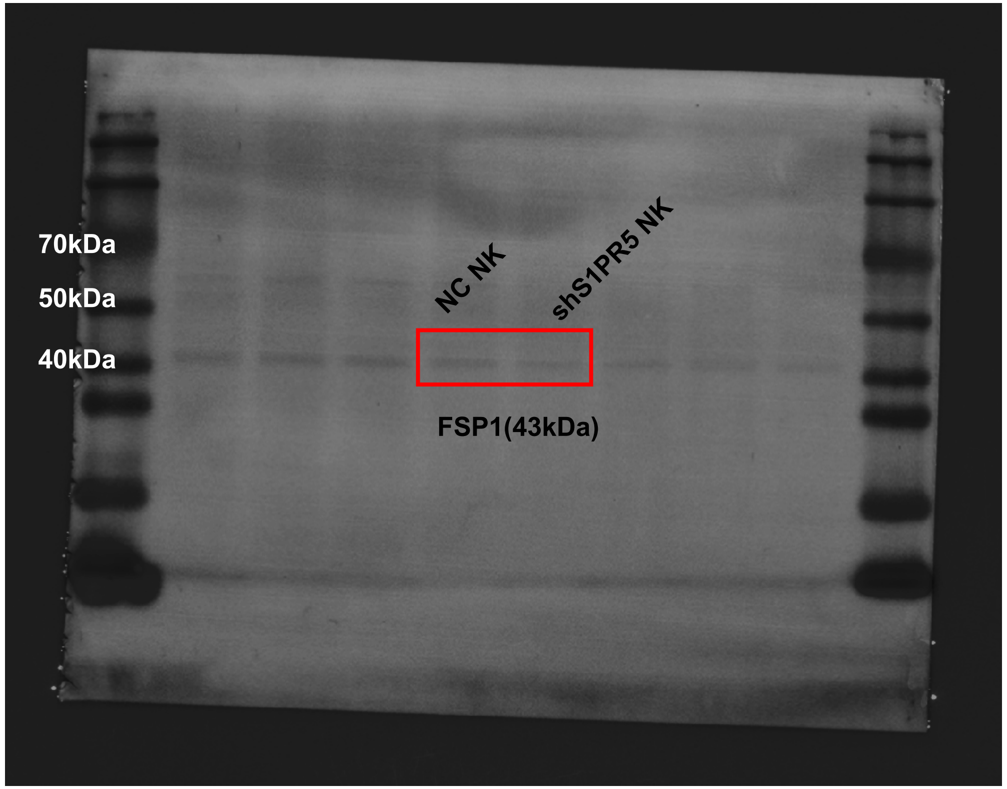

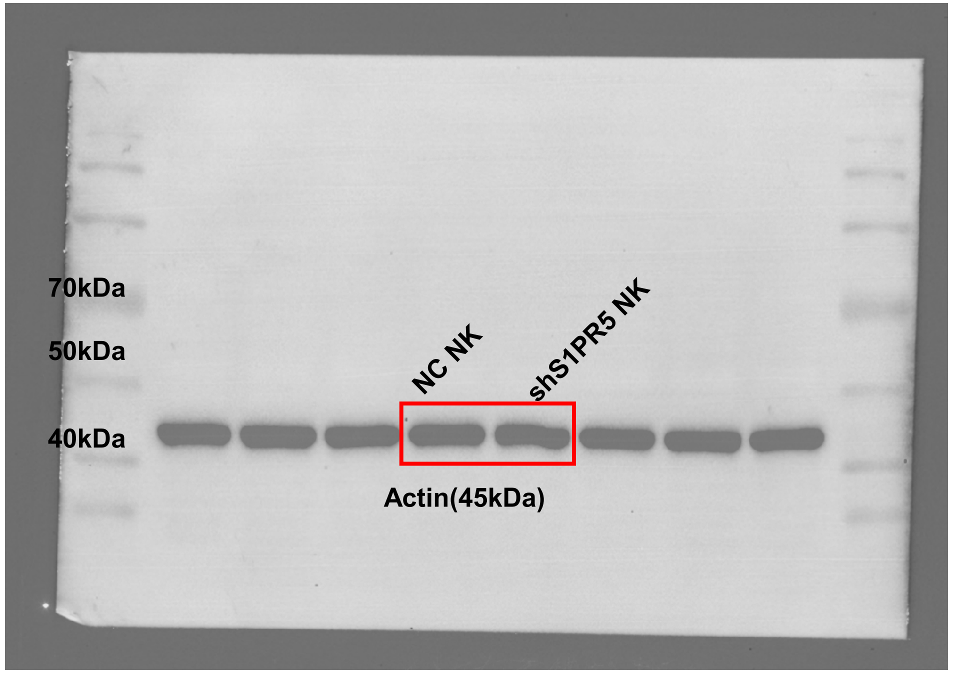

Supplement: Supplementary file 4 — Source Data [file 41467_2026_71792_MOESM4_ESM.zip › source data/wb source data.pdf]
